# Fortify the defense frontline: MAPKs phosphorylate receptor-like cytoplasmic kinase to maintain plant resistance in soybean

**DOI:** 10.1007/s44154-024-00164-y

**Published:** 2024-04-12

**Authors:** Lu Rui, Wei Wang

**Affiliations:** 1 College of Biology and Food Engineering, Chongqing Three Gorges University, Chongqing, 404120 China; 2 State Key Laboratory of Ecological Control of Fujian-Taiwan Crop Pests, Key Laboratory of Ministry of Education for Genetics, Breeding and Multiple Utilization of Crops, Plant Immunity Center, Fujian Agriculture and Forestry University, Fuzhou, 350002 China

**Keywords:** Plant immunity, Soybean, Nematode, Mitogen-activated protein kinase (MAPK), Receptor-like cytoplasmic kinase (RLCK)

## Abstract

Mitogen-activated protein kinase (MAPK) activation is one of the significant immune events that respond to pathogens in plants. A MAPK cascade often contains a MAPK kinase kinase (MAPKKK), a MAPK kinase (MAPKK/MKK), and a MAPK. The well-characterized MAPK cascade, to date, is the MAPKKK3/4/5-MKK4/5-MPK3/6 module. Soybean cyst nematodes (SCN) is one of the most devastating soybean pathogens. However, the early immune components contributing to soybean resistance to SCN and the role of the MAPK cascade in the soybean–SCN interaction remain unclear. A recent study published in Plant Cell discovered that GmMPK3/6 phosphorylates a receptor-like cytoplasmic kinase (RLCK), CDG1-LIKE1 (GmCDL1), and maintains the stability of GmCDL1 in soybean. Remarkably, GmCDL1 enhances GmMPK3/6 activation and resistance to SCN by phosphorylating GmMAPKKK5 and activating the GmMAPKKK5-GmMKK4-GmMPK3/6 cascade. In addition, two L-type lectin receptor kinases (LecRKs), GmLecRK02g and GmLecRK08g, are involved in the GmCDL1 function after the perception of SCN. taken together, this study not only discovers a complete early immune pathway that responds to SCN infection in soybean, but also reveals a molecular mechanism by which plants maintain the activation of the MAPK cascade and resistance.

The mitogen-activated protein kinase (MAPK) signaling pathway is a conserved signaling module in eukaryotes that plays essential roles in many biological processes, such as growth, development, and abiotic/biotic stress responses both in animals and plants (Sun & Zhang 2022). A MAPK cascade consisting of a MAPK kinase kinase (MAPKKK), a MAPK kinase (MAPKK/MKK), and a MAPK can be activated through phosphorylation by the plant membrane-localized pattern recognition receptor (PRR) complex upon the perception of extracellular pathogen-associated molecular patterns (PAMPs) or damage-associated molecular patterns (DAMPs) (Wu & Wang 2024). In *Arabidopsis*, the MEKK1-MKK1/2-MPK4 module and the MAPKKK3/4/5-MKK4/5-MPK3/6 module have been extensively studied. However, the molecular mechanism of MAPK cascade activation in soybean (*Glycine max*) has barely been elucidated, particularly in terms of the interaction between soybean and *Heterodera glycines Ichinohe* (soybean cyst nematode, SCN), one of the most devastating soybean pathogens.

It has been reported that the MEKK1-MKK1/2-MPK4 module negatively regulates *Arabidopsis* defense responses (Gao et al. 2008). Similarly, silencing the *AtMPK4* homolog in soybean, *GmMAPK4*, results in the overaccumulation of salicylic acid (SA) and enhanced resistance to soybean mosaic virus (SMV) and downy mildew (*Peronospora manschurica*). GmMPK4 interacts with and is phosphorylated by GmMKK1/2 (Liu et al. 2011). Moreover, *GmMKK1* silenced soybean plants exhibit immune-activating phenotypes (Xu et al. 2018), indicating that GmMKK1 synergizes with GmMPK4 to negatively regulate soybean immunity.

The PAMP/DAMP-induced activation of the MAPKKK3/4/5-MKK4/5-MPK3/6 module, which relies on upstream PRRs and receptor-like cytoplasmic kinases (RLCKs), is well established in the model plant *Arabidopsis*. Activated AtMPK3/6 directly phosphorylate their substrates to regulate resistance. Remarkably, to prolong resistance, AtMPK3/6 also maintain the activation of this cascade by phosphorylating MAPKKK5 in a positive feedback regulatory loop (Bi et al. 2018, Wang et al. 2023). Similarly, GmMKK4 interacts with and activates GmMPK6 via phosphorylation in soybean (Liu et al. 2014). Extensive research on MAPK cascade activation was recently published by Zhang et al., reveling the essential role of the GmMAPKKK5-GmMKK4-GmMPK3/6 cascade in the early stages of soybean and SCN interaction (Zhang et al. 2024).

In this study, the authors performed a phosphoproteome analysis under SCN infection and discovered peptides containing the potential MAPK phosphorylation motif pS/pTP, indicating the involvement of MAPKs in the soybean–SCN interaction. Subsequently, they found that GmMPK3/6 were activated after SCN infection. Suppression of MAPK activity and overexpression of GmMPK3/6 enhanced the susceptibility and resistance, respectively, of soybean plants to nematodes, indicating that MAPKs positively regulate soybean resistance to SCN. In addition, reactive oxygen species (ROS) accumulated and cell death occurred when GmMPK3/6 was overexpressed in soybean plants. Furthermore, GmMKK4 associated with GmMPK3/6, and overexpression of GmMKK4 activated GmMPK3/6, induced ROS bursts and cell death, and ultimately enhanced resistance to SCN. Although there is no direct evidence that GmMAPKKK5 activates GmMKK4 after SCN infection, these results indicate that the activation of the GmMAPKKK5-GmMKK4-GmMPK3/6 cascade is a key upstream signaling event in plant defense against nematode infection.

The MAPKKK3/4/5-MKK4/5-MPK3/6 cascade is activated by upstream RLCKs, such as the RLCK VII subfamily member AVRPPHB SUSCEPTIBLE1-LIKE 27 (PBL27) and the RLCK XII subfamily member BRASSINOSTEROID-SIGNALING KINASE1 (BSK1) (Rao et al. 2018, Yan et al. 2018). Interestingly, in the phosphoproteome analysis, Zhang et al. showed that the RLCK, CDG1-LIKE1 (GmCDL1), a homolog of the RLCK VII subfamily member AtCDL1 (also known as AtPBL7), was a substrate of GmMPK3/6. Full-length GmCDL1 was required for the interaction with GmMPK3/6. Wound treatment also enhanced GmCDL1 phosphorylation in a GmMPK3/6 dependent manner. Moreover, both *in vivo* and *in vitro* assays showed that GmMPK3/6 phosphorylated GmCDL1 at its Thr-372 residue. However, mutagenesis of Thr-372 neither affected the distribution of GmCDL1 nor interfered with the interaction between GmCDL1 and GmMPK3/6. Undeniably, the abundance of RLCK is critical for plant resistance activation. For example, the accumulation of BOTRYTIS‐INDUCED KINASE 1 (AtBIK1), a member of the RLCK VII subfamily, is fine-tuned by different E3 ubiquitin ligases. The plant U-box proteins AtPUB25/26 ubiquitinate and mediate the degradation of nonactivated BIK1 and negatively regulate plant immunity, whereas the E3 ubiquitin ligases RING-H2 FINGER A3A (AtRHA3A) and AtRHA3B monoubiquitinate the phosphorylated BIK1 and promote its release from the FLS2–BAK1 complex, ultimately positively regulating plant immunity (Ma et al. 2020, Wang et al. 2018). In the further study, the authors revealed that the protein levels of the phospho-mimetic variant GmCDL1^T372D^ were stable and that the protein levels of the phospho-dead variant GmCDL1^T372A^ were reduced, indicating that the phosphorylation of GmCDL1 at Thr-372 enhances its protein stability. Consistently, blocking the function of MAPKs with U0126, a MAPK pathway inhibitor, reduced the accumulation of GmCDL1. Moreover, the results of the MG132 treatment assay showed that GmCDL1 degradation was dependent on the 26S proteasome. However, further investigations are needed to identify the specific components that fine-tune GmCDL1. Furthermore, a reduction in GmCDL1 levels in soybean roots resulted in weakened GmMPK3/6 activation and increased susceptibility to SCN. However, the overexpression of GmCDL1 and GmCDL1^T372D^, but not GmCDL1^T372A^, enhanced GmMPK3/6 mediated resistance to SCN, indicating that GmCDL1 contributes to soybean resistance to SCN and that the phosphorylation of Thr-372 is required for its function.

The abovementioned results suggest that RLCK GmCDL is a substrate of GmMPK3/6 and that GmMPK3/6 positively regulate soybean resistance by phosphorylating and stabilizing GmCDL1. However, RLCK proteins have been reported to activate the MAPK cascade by phosphorylating MAPKKKs, such as AtMAPKKK5 (Yan et al. 2018). Therefore, the authors also examined and confirmed that GmCDL1 interacted with and phosphorylated GmMAPKKK5, indicating that GmCDL1 positively modulates soybean resistance to SCN by regulating the activation of the GmMAPKKK5-GmMKK4-GmMPK3/6 cascade. Activated GmMPK3/6 in turn phosphorylate and stabilize GmCDL1, leading to prolonged activation of MAPK cascades and amplification of disease resistance, which reveals a central mechanism by which plants regulate the complete activation of the MAPK cascade.

However, which PRR functions upstream of GmCDL1 in recognizing the SCN has not yet been determined. In the present study, Zhang et al. performed liquid chromatography–tandem MS (LC-MS/MS) and identified two L-type lectin receptor kinases (LecRKs), GmLecRK02g and GmLecRK08g, which interacted with and phosphorylated GmCDL1. Moreover, GmLecRK02g and GmLecRK08g positively regulated MAPK activation and SCN resistance. The potential phosphorylation sites mediated by GmLecRK08g in GmCDL1 were Ser-234 and Thr-235. These residues were required for GmCDL1-mediated MAPK activation after wound treatment and were conserved in AtBIK1, corresponding to Ser-236 and Thr-237, suggesting that both the Ser-234 and Thr-235 residues may play important roles in regulating the activation and function of many RLCKs.

In summary, although the specific nematode-associated molecular patterns (NAMPs) recognized by GmLecRK02g and GmLecRK08g and the downstream components corresponding to GmMPK3/6 activation were not identified in this study, Zhang and his colleagues established an early and complete immune pathway, GmRKs-GmCDL1-GmMAPKKK5-GmMKK4-GmMPK3/6, that responds to soybean cyst nematode infection and damage signals. In this pathway, GmLecRK02g and GmLecRK08g sense extracellular signals and phosphorylate GmCDL1 at Ser-234 and Thr-235 to activate GmCDL1 and the downstream MAPK cascade. Remarkably, activated GmMPK3/6 in turn phosphorylates GmCDL1 at Thr-372 to prevent the degradation of GmCDL1 via the 26S proteasome system, resulting in further activation of the MAPK cascade and enhanced resistance to SCN (Fig. 1). Taken together, these results highlight the molecular mechanism by which plants stabilize and maintain complete activation of the MAPK cascade in the soybean–SCN interaction.

**Fig. 1:**
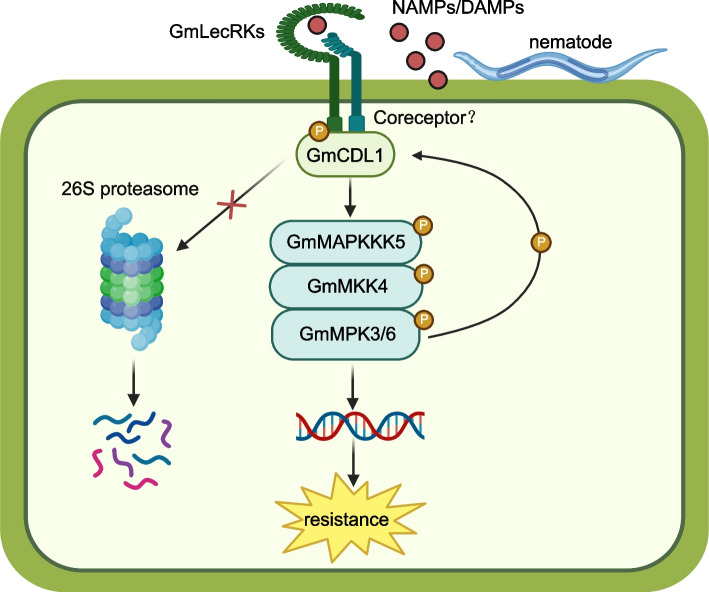
The regulation of MAPK cascade activation in the soybean–SCN interaction. GmLecRKs receive unknown extracellular NAMPs/DAMPs and phosphorylate GmCDL1 to promote its activation and dissociation. GmCDL1 positively regulates immune responses by activating the GmMAPKKK5-GmMKK4-GmMPK3/6 cascade. Activated GmMPK3/6 in turn phosphorylate GmCDL1 to maintain its stability, leading to further amplification of disease resistance

## Data Availability

Not applicable.

## Abbreviations

SCN: Soybean cyst nematode
MAPKKK: Mitogen-activated protein kinase kinase kinase
MAPKK/MKK: Mitogen-activated protein kinase kinase
MAPK: Mitogen-activated protein kinase
P/D/NAMP: Pathogen/Damage/Nematode-associated molecular pattern
PRR: Pattern recognition receptor
ROS: Reactive oxygen species
RLCK: Receptor-like cytoplasmic kinase
LecRKs: L-type lectin receptor kinases
